# Meet the authors: Hanchuan Peng, Peng Xie, and Feng Xiong

**DOI:** 10.1016/j.patter.2023.100912

**Published:** 2024-01-12

**Authors:** Hanchuan Peng, Peng Xie, Feng Xiong

**Affiliations:** 1 SEU-ALLEN Joint Center, Institute for Brain and Intelligence, Southeast University, Nanjing, Jiangsu 210096, China

## Abstract

In a recent paper at *Patterns*, Hanchuan Peng, Peng Xie, and Feng Xiong from Southeast University describe a deep learning method to characterize complete single-neuron morphologies, which can discover neuron projection patterns of diverse cells and learn neuronal morphology representation. In this interview, the authors shared the story behind the paper and their research experience.

This interview is a companion to these authors’ recent paper, “DSM: Deep sequential model for complete neuronal morphology representation and feature extraction.”^1^

## Main text

### What would you like to share about your research background?

**Figure:**
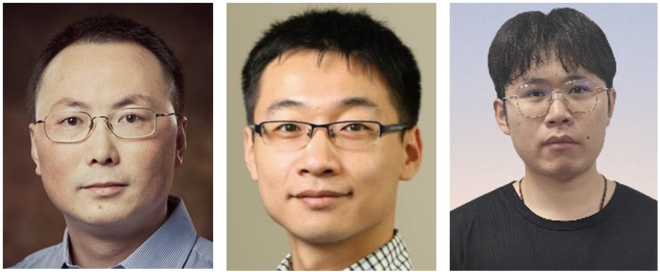
Figure: From left to right: Hanchuan Peng, Peng Xie, and Feng Xiong

**Hanchuan Peng:** I am deeply interested in the intersection of AI and brain science, and my team is actively exploring this dynamic relationship. Our research focuses on unraveling the organizational principles of biological neuronal networks to inspire the design of artificial neural networks. We also delve into correlating the computational adaptability of artificial neural networks with the firing patterns of individual neurons and collective behaviors in neuronal networks, enhancing our understanding of the interplay between artificial and biological systems. Additionally, we investigate the mechanisms governing information storage, transfer, and inheritance in both biological and artificial neuronal networks, seeking parallels and distinctions. Leveraging AI techniques, we aim to comprehend the structure and functions of biological neuronal networks on a whole-brain scale, pushing the boundaries of understanding in the field. Overall, our research represents a holistic exploration of the symbiotic relationship between AI and brain science, covering organizational principles, computational adaptability, information dynamics, and advanced AI applications for deeper insights into the complexities of the brain.

**Peng Xie:** My research background is in bioinformatics and pattern recognition. I am interested in utilizing computational tools for deciphering information underlying complex biological patterns. My pursuit of this direction initiated when I studied regulatory DNA sequences as a PhD student. During the past few years, I have designed and applied machine learning approaches to seek hidden representations of biological big data and driving regulators of biological processes, especially cell fate determination.

**Feng Xiong:** I am pursuing a PhD in the field of brain science and artificial intelligence at the SEU-ALLEN Joint Center of Institute for Brain and Intelligence at Southeast University. I joined the Center as a postgraduate student in 2019, and my research work started there. In the first 2 years, I participated in building an automatic neuron registration pipeline and registering 1,741 neuron reconstructions to a standard mouse brain template. The registration data were further used by our teams in two major projects about single-neuron morphological analysis and registration.^2^^,^^3^ In 2021, I started to develop deep learning models to characterize neuron morphologies and classify neuron reconstructions. Since then, I have kept learning new programming skills and data science techniques and applying these methods in various fields. The deep sequential model (DSM) manuscript finally came out and was published in *Patterns*.^1^ In 2022, I participated in morphology analysis of human neurons (Han et al.^4^). The analysis of neuron morphology indicates the diversity of human neurons across brain regions. Now, I focus on projects about single-neuron connectivity and single-cell transcriptomic analysis.

### Let’s start with the first author. What is the definition of data science in your opinion? What is a data scientist? Do you self-identify as one?

**FX:** I think that data science is a subject that characterizes scientific data and discovers the inner patterns to solve scientific problems. It requires not only the understanding of data to define proper features but also sufficient knowledge to establish models to analyze data. Therefore, data scientists are those familiar with domain knowledge in specific scientific fields and who also have experience in characterizing data and building analysis models. At the current stage, I don’t identify myself as a data scientist. I am a beginner in data science, and there is still a lot to learn.

### How do you keep up to date with advances in both data science techniques and brain science?

**FX:** The development of new techniques has provided more and more ways to study scientific problems, which also brings more challenges to data analysis. It is important to keep learning new skills and stay up to date with the new knowledge in the field. I use media and read papers to access these advancements. In general, I first use social media to track new technologies and then read relevant papers that I am interested in. It is also necessary to check their codes and application programming interfaces (APIs) for deep understanding and further development.

### Did you encounter any particular difficulties, or were there any specific challenges about data, data management, or FAIR data sharing that you dealt with? How did you overcome them? Can others use the solutions you used to overcome these challenges?

**FX:** Data quality is essential for downstream analysis. Therefore, it is always the first step to clean data and perform quality checks for data analysis. For this work^1^ in *Patterns*, a series of preprocessing steps were taken to ensure data quality. For example, we ensured the correctness of neuron topology and repaired the multi-bifurcation of neuron structure. We sampled the neuron reconstruction to reduce the computation scale and more. We also accessed two external neuron reconstruction datasets for method comparison. The annotation of neurons in these two datasets is different from ours, so we manually annotated lots of cells for our comparison.

### What’s next for the project? What’s next for you?

**FX:** In this work,^1^ we explored the diversity of whole-brain projection patterns of mouse neurons and utilized the characteristics to define cell types. It reveals the connectivity of the mouse brain. Our next project is further studying the connectivity of single neurons. The projection of cells reflects the potential connectivity of source brain regions and target brain regions, but in fact, connections of cells from these brain regions are real building blocks for brain circuits. I will continue to explore this direction using my data science skills, and I will keep learning new techniques to be a good data scientist.

### Dr. Peng and Dr. Xie, how did this project you wrote about come to be?

**HP:** In our recent work,^2^^,^^4^^,^^5^ we’ve curated several of the most extensive databases capturing the neuron morphology in brains of different species, such as mouse,^2^ human,^4^ as well as insects and many other species.^5^ Analyzing such rich datasets, we instinctively sought to categorize neurons based on their morphological features. An observation emerged: previous methodologies typically overlooked incorporating projection patterns in morphological feature clustering. In response, we pioneered a novel approach,^1^ employing AI techniques to precisely model the projection patterns of neurons. This innovative integration not only advances the accuracy of our analyses but also represents a significant departure from conventional methods. By incorporating the intricate details of neuronal projection patterns, our approach showcases the potential of AI to revolutionize the study of neuronal morphology in mammalian brains.

**PX:** I’d like to add some details about the idea of incorporating the projection patterns for representing neuronal morphology. By the time we started this work, AI-based sequence models achieved state-of-the-art performance in several tasks, e.g., the transformer model in natural language processing. The long-projecting morphology of a neuron can be naturally represented by sequence-type data structures, which is a suitable research object for such AI models. The information of the neuronal projecting path is also complementary to previous metrics people used for morphological characterization. So we decided this must be a worthy attempt.

### Can you tell us more about your team? Where is the team currently based, and how long have you been there?

**HP:** Our core team is currently located in Nanjing, an enchanting city in the eastern part of China. Over the past 5 years, we have proudly developed our research center into a thriving Open Science hub. This hub serves as a welcoming space for international researchers from diverse countries, fostering collaboration and knowledge exchange.

### An Open Science hub? What are your thoughts of open science?

**HP:** Open science serves as a foundational framework in our research, facilitating transparent communication with both colleagues and the global community. Its indispensability is unquestionable. However, it is imperative to recognize that the adoption of open science comes with associated responsibilities and costs. While the benefits of open science are substantial, it’s crucial to emphasize that it is not a cost-free endeavor. To derive maximum value from open science, a dual commitment is required: first, ensuring the integrity of the data contributed and second, preventing the misuse or abuse of the data consumed. In essence, the true potential of open science can only be realized when accompanied by a commitment to maintaining the highest standards of data integrity and responsible data usage. This dual commitment ensures that the collaborative nature of open science continues to thrive, fostering a culture of trust and reliability in the scientific community.

### What kind of atmosphere do you look to foster in your team? Is there anything you try to replicate or avoid from your own experiences or that you have learned over the years?

**HP:** Embracing an open science, big science, and team science philosophy, our approach to studying brain science and AI draws inspiration from my previous roles at the Allen Institute for Brain Science and the Howard Hughes Medical Institute’s Janelia Research Campus. Our commitment to openness has proven to be a powerful catalyst for advancing scientific inquiry, reflecting a belief cultivated through experiences that being transparent and collaborative is pivotal for fostering excellence in research.
